# Late Sodium Current Inhibitors as Potential Antiarrhythmic Agents

**DOI:** 10.3389/fphar.2020.00413

**Published:** 2020-04-20

**Authors:** Balázs Horváth, Tamás Hézső, Dénes Kiss, Kornél Kistamás, János Magyar, Péter P. Nánási, Tamás Bányász

**Affiliations:** ^1^Department of Physiology, Faculty of Medicine, University of Debrecen, Debrecen, Hungary; ^2^Faculty of Pharmacy, University of Debrecen, Debrecen, Hungary; ^3^Division of Sport Physiology, University of Debrecen, Debrecen, Hungary; ^4^Department of Dental Physiology and Pharmacology, Faculty of Dentistry, University of Debrecen, Debrecen, Hungary

**Keywords:** voltage gated sodium channel, late sodium current, arrhythmias, antiarrhythmic drugs, sodium channel inhibitors

## Abstract

Based on recent findings, an increased late sodium current (I_Na,late_) plays an important pathophysiological role in cardiac diseases, including rhythm disorders. The article first describes what is I_Na,late_ and how it functions under physiological circumstances. Next, it shows the wide range of cellular mechanisms that can contribute to an increased I_Na,late_ in heart diseases, and also discusses how the upregulated I_Na,late_ can play a role in the generation of cardiac arrhythmias. The last part of the article is about I_Na,late_ inhibiting drugs as potential antiarrhythmic agents, based on experimental and preclinical data as well as in the light of clinical trials.

## Introduction

**Figure 1:**
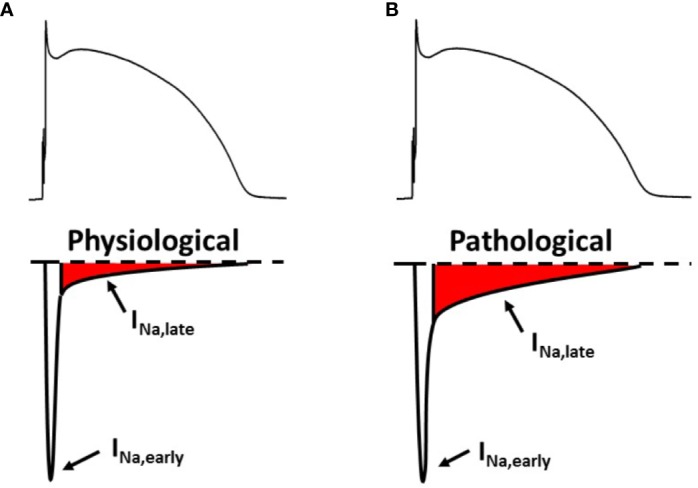
The early and the late component of the sodium current under physiological **(A)** and pathological **(B)** conditions. Upper panels: membrane potential; lower panels: sodium current. I_Na,early_, early (peak) component of the sodium current; I_Na,late_, late (sustained) component of the sodium current.

During the non-pacemaker action potential (AP) in the heart, depolarization of the cell membrane opens voltage gated sodium channels (Na_v_) for a short period of time (Scanley et al., 1990; Mitsuiye and Noma, 2002) giving rise to the early sodium current peak (I_Na,early_). This I_Na,early_ causes the upstroke of the non-pacemaker AP. Through the course of the AP Na_v_ channels may recover from inactivation and reopen, generating a sustained current component, called late sodium current (I_Na,late_). I_Na,late_ flows throughout the plateau phase of the AP therefore it significantly contributes to AP morphology, even though its magnitude is only a fraction of I_Na,early_ (**Figure 1A**).

If I_Na,late_ is increased, it might play a pathophysiological role in acquired cardiac diseases (**Figure 1B**) such as myocardial ischemia (Maier and Sossalla, 2013) and heart failure (Coppini et al., 2013; Pourrier et al., 2014). In the cardiomyocytes, an upregulated I_Na,late_ hinders repolarization and causes a larger sodium entry, therefore increasing intracellular sodium concentration ([Na^+^]_i_). An increased [Na^+^]_i_, in turn, leads to a larger intracellular calcium content. These factors together can possibly cause contractile dysfunction (Sossalla et al., 2011), disturbed myocardial energetics (Liu and O'Rourke, 2008) and cardiac arrhythmias (Antzelevitch et al., 2014).

## Electrophysiological Identification of I_Na,late_

Mammalian cardiac cells express a wide variety of Na_v_ isoforms, differing in unit conductance, voltage sensitivity, kinetics, and drug sensitivity. In the majority of cardiac tissues, the dominant isoform of the pore-forming subunit is Na_v_1.5, which is relatively insensitive to the sodium channel toxin tetrodotoxin (TTX) (Gellens et al., 1992; Catterall et al., 2005). Many of the TTX-sensitive (“non-cardiac”) Na_v_ channels (Na_v_1.1, Na_v_1.2, Na_v_1.3, Na_v_1.4, and Na_v_1.6) are also shown to be present in cardiac tissue (Maier et al., 2002; Haufe et al., 2005; Valdivia et al., 2005; Biet et al., 2012; Yang et al., 2012). In nodal tissue Na_v_1.1 and Na_v_1.6 are expressed in the largest quantities. Besides the pore-forming subunit, four auxiliary subunits (ß_1_, ß_2_, ß_3_, and ß_4_) and certain scaffolding proteins also participate in building up the whole complex, which also attaches to the cytoskeleton. These molecules can interact with each other and may modify the kinetics and voltage dependence of the actual channel (Malhotra et al., 2001).

Mechanisms that are discussed in the followings may contribute to the profile of I_Na,late_ during the AP. Understanding these mechanisms better might be helpful in developing new antiarrhythmic therapeutic strategies targeting I_Na,late_.

### I_Na,late_ Is Underlain by Different Channel Gating Modes

At the resting membrane potential, the vast majority of Na_v_1.5 channels are in their closed state. Upon depolarization, Na_v_1.5 channels open up within 1–2 ms after which they inactivate rapidly (Scanley et al., 1990; Mitsuiye and Noma, 2002). This produces I_Na,early_ and the upstroke of the non-pacemaker cardiac AP. During a sustained depolarization, Na_v_1.5 channels can reopen with a small probability. In ventricular myocytes, three modes of Nav1.5 channel activity have been characterized in single-channel experiments: transient mode (TM), burst mode (BM), and late scattered mode (LSM) (Maltsev, 2006).

I_Na,early_ is mainly the result of TM activity, while BM and LSM are responsible for the sustained sodium current, I_Na,late_ (**Figure 1A**). The magnitude of the sustained current component is only about 0.5–1 % of I_Na,early_ measured 50 ms after the onset of the depolarizing pulse (Maltsev, 2006). During a sustained depolarization BM openings rapidly decline in the first tens of milliseconds therefore leaving LSM as the gating mode being mainly responsible for I_Na,late_ toward the end of the plateau phase.

Mutations of the channel protein and certain diseases can change the contribution of different Na_v_1.5 channel activity patterns to the macroscopic current, therefore increasing I_Na,late_ (Bezzina et al., 1999; Valdivia et al., 2005; Wu et al., 2006; Maltsev et al., 2007; Maltsev and Undrovinas, 2008; Song et al., 2008; Maltsev et al., 2009; Xi et al., 2009; Guo et al., 2010; Trenor et al., 2012) (**Figure 1B**). Apparently, each gating mode has a distinct drug sensitivity or drug affinity as well (Belardinelli et al., 2004; Ravens et al., 2004; Belardinelli et al., 2006). Based on this, selective pharmacological targeting of certain gating modes might have potential antiarrhythmic and/or cardioprotective effects (Belardinelli et al., 2006; Hoyer et al., 2011; Morita et al., 2011).

### Window Sodium Current

The voltage dependence of the steady state activation and inactivation of most Na_v_ channels overlaps with each other (Zaza and Rocchetti, 2013). This overlap provides a voltage range (“window”) where inactivated Na_v_ channels are able to recover from inactivation and then might reopen. When the actual membrane potential falls within this “window” of overlap, a sustained current is evoked. Under physiological circumstances this “window current” mechanism likely plays a limited role in I_Na,late_, because the Na_v_1.5 voltage “window” is around −70 mV, falling quite far from the AP plateau. Additionally, in the window voltage range, the current density is less than 5 % of the maximum current density in healthy myocytes (Maltsev et al., 1998; Wang et al., 2002; Liu et al., 2007). Hence, the “window current” mechanism is unlikely to be a major determinant of I_Na,late_ in healthy myocytes. Mutations of channel proteins or altered regulation in certain diseases may shift either the steady-state activation or inactivation curves of Na_v_ channels to significantly change this voltage window, therefore increasing I_Na,late_ under these pathological conditions (Wang et al., 1996; Ruan et al., 2009).

### Non-Equilibrium Channel Gating

During the AP of cardiac myocytes, the membrane potential changes continuously. Na_v_ channels are incorporated into this dynamic system. It has been proposed by Clancy et al. (2003) that the voltage “history” of the cell membrane can modulate the transition between Na_v_ channel states, termed “non-equilibrium gating”. As a result, recovery from inactivation is also modulated by the dynamics of voltage change. The theory is supported by experimental data showing that the application of repolarizing voltage ramps or AP shape voltage commands evoke a larger I_Na,late_ compared to conventional square pulses or model simulations where “non-equilibrium gating” is not incorporated into the numerical model (Clancy et al., 2003; Magyar et al., 2004; Horvath et al., 2013).

### Non-Cardiac Sodium Channel Isoforms in the Heart

Epilepsy (Alekov et al., 2000; Akalin et al., 2003) and certain skeletal muscle diseases (Komajda et al., 1980; Pereon et al., 2003) has been associated with pathological ECG recordings. Therefore it seemed possible that non-cardiac sodium channel mutations might cause electrical alterations in the heart. Later, Na_v_1.1, Na_v_1.2, Na_v_1.3, Na_v_1.4, Na_v_1.6, and Na_v_1.8 isoforms have been identified in cardiac tissue (Maier et al., 2002; Haufe et al., 2005; Valdivia et al., 2005; Biet et al., 2012; Yang et al., 2012). Based on the findings of *Biet et al*., as much as 44 % of I_Na,late_ is due to non-cardiac sodium channels (Biet et al., 2012) in canine ventricular cardiomyocytes. Furthermore, *Yang et al*. have shown that in mice and rabbit the TTX-resistant Na_v_1.8 provides a substantial amount of I_Na,late_ (Yang et al., 2012). Based on these experimental data, isoform specific sodium channel modulators might provide a valid approach in pharmacological antiarrhythmic therapy (See *Non-Cardiac Sodium Channel Inhibitors as Potential Antiarrhythmic Agent* for further details).

## Role of I_Na,late_ in Cardiac Physiology

### Role of I_Na,late_ in Cardiac Electrical Activity

Contribution of I_Na,late_ to cardiac APs was questioned because of its small density. However, the plateau phase of the cardiac AP is shaped by a delicate balance between minuscule inward and outward current fluxes. Therefore even a small change in these currents may significantly alter the duration of the AP (Horvath et al., 2006). Inhibition of I_Na,late_ substantially shortens the cardiac AP in the conductive system (Coraboeuf et al., 1979) and in ventricular cells (Kiyosue and Arita, 1989) as well, indicating that I_Na,late_ significantly contributes to determining the duration of the non-pacemaker AP in cardiac myocytes. Recent AP voltage clamp experiments show that the density of I_Na,late_ is of similar magnitude as the major potassium currents in guinea pig (Horvath et al., 2013) and rabbit (Hegyi et al., 2018) ventricular myocytes. There is a characteristic interspecies difference in the shape of I_Na,late_ as shown in the case of guinea pig, canine, and human ventricular myocytes (Horvath et al., 2020).

The sustained sodium current is also an important factor in determining electrophysiological properties of sinoatrial node cells (Maier et al., 2003; Lei et al., 2004). Tetrodotoxin, applied in lower than 1 µM concentrations, reduces the rate of spontaneous depolarization in sinoatrial node cells (Huang et al., 2015), clearly indicating that non-cardiac Na_v_ isoforms also contribute to cardiac automaticity.

Cardiac Purkinje cells have the largest rate-dependence of their AP duration (APD) among cardiomyocytes with fast response APs. Purkinje cell APs are longer at lower stimulation rates, while shorter at higher rates than APs of ventricular cells. It has been shown that I_Na,late_ contributes to this feature by possessing much slower decay and recovery kinetics in Purkinje cells than in ventricular cells. As a result Purkinje cell I_Na,late_ is significantly larger at low heart rates, while smaller at high heart rates compared to ventricular cells. This unique feature predisposes Purkinje cells to serve as triggers in generating arrhythmias (Li et al., 2017).

I_Na,late_ plays a role in forming the atrial AP as well (Burashnikov and Antzelevitch, 2013; Luo et al., 2014). I_Na,late_ is expected to be larger in atria than in ventricles because I_Na,_ _early_ density is greater in atrial cells under similar conditions (Li et al., 2002; Burashnikov et al., 2007), suggesting a higher sodium channel expression in atrial cells. On the other hand, an overall more positive membrane potential, and a more negative steady-state inactivation voltage of the sodium current (Li et al., 2002; Burashnikov et al., 2007) in the atrial cells reduce the availability of the sodium channels (Burashnikov and Antzelevitch, 2008). In one set of experiments by Luo et al. maximum I_Na,late_ density has been reported to be greater in rabbit left atrial myocytes than in ventricular cells (Luo et al., 2014) and in a different investigation the two cell types seemed to be similar in this matter (Persson et al., 2007). APs are shorter in the atria compared to the ventricles reducing the amount of Na^+^ influx through I_Na,late_ in the former (Burashnikov and Antzelevitch, 2013).

### I_Na,late_ Plays a Significant Role in the Sodium Homeostasis of Cardiomyocytes

[Na^+^]_i_ is set by a dynamic equilibrium of the influx of Na^+^ into the cell and efflux of Na^+^ to the interstitial space. The [Na^+^]_i_ of non-paced ventricular myocytes is around 4–8 mM in guinea-pig, rabbit, and canine; and about twice as high in rat and mouse (9–14 mM) (Despa and Bers, 2013). In non-paced human myocytes [Na^+^]_i_ is thought to be in the 4–10 mM range.

Na^+^ can enter into the cell through Na^+^ channels, Na^+^/Ca^2+^ exchanger (NCX) and Na^+^/H^+^ exchanger (NHE). Na^+^ leaves the cell mainly via the Na^+^/K^+^ pump (NKP), but the reverse mode NCX is also responsible for a moderate Na^+^ efflux during the first few milliseconds of the cardiac AP. Furthermore, Na^+^/HCO_3_^−^ cotransport, Na^+^/Mg^2+^ exchange, and Na^+^/K^+^/2Cl^−^ cotransport can play a role in the sodium homeostasis of cardiomyocytes to a small extent (Despa and Bers, 2013). It also has to be mentioned that Na^+^ concentrations between the cytosol and intracellular organelles are continuously balanced.

Upon pacing, [Na^+^]_i_ increases with increasing stimulation frequency, caused by the larger Na^+^ entry through Na^+^ channels and NCX. In paced, single cardiac cells approximately 25 % of the Na^+^ entry is mediated by Na_v_ channels (Despa and Bers, 2013). The Na^+^ entry through Na_v_ channels is about equally distributed between I_Na,early_ and I_Na,late_ (Makielski and Farley, 2006; Zaza and Rocchetti, 2013; Despa and Bers, 2013; Shryock et al., 2013), however this contribution can change at different heart rates (see *Heart Rate and AP Duration Influences I_Na,late_* for details). The higher Na^+^ influx into paced cells is matched by an increased efflux through an elevated NKP activity. This is mainly caused by the increased [Na^+^]_i_ itself, but nitric oxide-, and phospholemman-dependent mechanisms can also add to this effect (Despa and Bers, 2013).

### Na^+^ and Ca^2+^ Homeostasis Is Linked in Cardiomyocytes

#### The Direct Connection Between Na^+^ and Ca^2+^ Homeostasis: Na^+^/Ca^2+^ Exchanger

The NCX is a secondarily active transporter that carries 1 Ca^2+^ and 3 Na^+^ at the same time (Janvier and Boyett, 1996; Fujioka et al., 2000; Sipido et al., 2007; Despa and Bers, 2013; Ginsburg et al., 2013). The NCX function is determined by the relation of the actual membrane voltage and the sum of the actual electrochemical gradients of Ca^2+^ and Na^+^. The main role of NCX is to remove Ca^2+^ from the cells by utilizing the potential energy present in the form of Na^+^ gradient (“forward mode”). Besides this mode, in the first few milliseconds of the AP, NCX mediates Na^+^ extrusion from the cell and Ca^2+^ entry into the cytosol (“reverse mode”).

#### I_Na,late_ Facilitates Ca^2+^ Influx via L-Type Calcium Channels

Being an inward current, I_Na,late_ depolarizes the membrane, causing an increased membrane potential throughout the plateau phase and a longer AP. The more time the membrane spends in a depolarized state (above +40 mV) the higher the possibility that L-type calcium channels can open or re-open. It is well documented with AP voltage clamp technique that the L-type calcium current is flowing throughout the AP plateau (Linz and Meyer, 1998; Linz and Meyer, 2000; Banyasz et al., 2003; Fulop et al., 2004; Banyasz et al., 2012). Therefore a longer AP inevitably results in a larger Ca^2+^ entry to the myocyte.

### Heart Rate and AP Duration Influences I_Na,late_

Heart rate determines the magnitude of I_Na,late_. Like many electrophysiological characteristics of cardiac cells (Banyasz et al., 2009), I_Na,late_ is reverse-rate dependent, so the faster the stimulation rate the smaller the current density will be (Nagatomo et al., 2002; Wu et al., 2011). However, with increasing heart rate the density of I_Na,early_ and maximum rate of depolarization during the AP upstroke (V_max_; an AP parameter determined by I_Na,early_) does not decrease that much (Nagatomo et al., 2002). This is because recovery of I_Na,late_ is much slower than I_Na,early_ (Carmeliet, 2006). At higher heart rates this feature of the two sodium current components also results in a decreasing contribution of I_Na,late_ to the overall Na^+^ influx. Under these conditions, the more frequent AP upstrokes cause a greater Na^+^ entry through I_Na,early_, and there is a reduction of I_Na,late_ density because of the very slow I_Na,late_ recovery kinetics. Moreover, rate-dependent changes of the AP length also influence Na^+^ entry. At high heart rates APs are shorter, therefore I_Na,late_ is active for a shorter time, accounting for a further reduction of Na^+^ influx through the already smaller I_Na,late_. At the same time, extrusion of Na^+^ by the NKP is reduced at high pacing rates (Despa and Bers, 2013) leading to a rate-dependent [Na^+^]_i_ loading in isolated cells. It must also be noted that this phenomenon is largely offset or may not occur at all during β-adrenergic stimulation because it augments NKP activity through phospholemman (Cheung et al., 2010).).

As it is described in the previous section, APD influences I_Na,late_: the shorter the AP the smaller the Na^+^ flux through I_Na,late_ is. Therefore under any conditions that result in a shorter AP the contribution of I_Na,late_ to the overall Na^+^ influx will be smaller. This fact, together with significant differences in heart rate underlies differences in I_Na,late_ between species having short APs (e.g.: rats or mice) and long APs (guinea pig, rabbit, pig, human, etc.). In rats and mice both I_Na,late_ and Na^+^ influx driven by I_Na,late_ should be much smaller than in species having long APs.

### Modulation of I_Na,late_

#### Cytosolic Ca^2+^ Modulates I_Na,late_ in a Complex Way

Ca^2+^ is the key player in the excitation-contraction coupling of cardiac cells and it also regulates many other cellular functions including sarcolemmal transport mechanisms. Na_v_ channels are regulated by the individual and cooperative actions of Ca^2+^, calmodulin (CaM), and Ca^2+^-CaM dependent protein kinase II (CaMKII) as well (Bers and Grandi, 2009; Maier, 2011; Scheuer, 2011). Signaling through the Ca^2+^—CaM—CaMKII pathway is thought to facilitate the sodium current, especially I_Na,late_ (Maltsev et al., 2008; Maltsev et al., 2009; Bers and Grandi, 2009).

#### Na_v_ Channels, Ca^2+^ and CaM

Motifs with Ca^2+^ binding (EF hand) as well as CaM binding (IQ motifs) capabilities are present in the Na_v_1.5 channel structure. Some groups have shown that Ca^2+^ alone can regulate sodium channels (Wingo et al., 2004), while other results support that Ca^2+^ is not capable of regulating Na_v_ channels directly; the regulation is mediated via Ca^2+^-CaM complex (Tan et al., 2002; Kim et al., 2004). Besides the exact regulatory mechanism, the general agreement is that when Ca^2+^ is elevated the SSI curve shifts toward more positive voltages (Sarhan et al., 2012), although this is a largely negligible effect at physiologically relevant Ca^2+^ concentrations in wild type channels. However, under conditions when Ca^2+^ or CaM sensing regions are mutated or when the Ca^2+^ sensitivity of Na_v_ channels are severely altered, diverse functional disturbances may arise leading to an increased I_Na,late_.

#### Ca^2+^-CaM Dependent Protein Kinase II (CaMKII)

Besides the direct regulation of Na_v_ channels, the Ca^2+^-CaM complex activates CaMKIIδ_C_ that also modulates these channels (Zhang and Brown, 2004; Anderson, 2005; Bers and Grandi, 2009). The active CaMKII is a Ser/Thr kinase that can phosphorylate Na_v_1.5 channels on at least three amino acid residues (Grandi and Herren, 2014). While there is an ongoing debate about the exact role of these phosphorylation sites in channel gating, all the studies agree on that activation of CaMKII increases I_Na,late_.

#### Complex Modulation by β-Adrenergic Stimulation

In a meticulous set of AP voltage clamp experiments, Hegyi et al. (Hegyi et al., 2018) showed how different downstream elements of the β-adrenergic pathway regulate I_Na,late_ in rabbit ventricular myocytes. Protein kinase A, CaMKII, Epac, nitrosylation, as well as reactive oxygen species (ROS) contributed to the upregulation of I_Na,late_ during different phases of the ventricular AP.

#### Cellular Metabolites

ROS and H_2_O_2_ increase I_Na,late_ (Song et al., 2004; Song et al., 2006; Sossalla et al., 2008). Some results suggest that CaMKII can be involved in I_Na,late_ facilitation observed in the presence of oxygen free radicals (Wagner et al., 2011), because ROS can also activate CaMKII (Erickson et al., 2008). See (Wagner et al., 2013) for a detailed review.

Acidosis also modulates Na_v_ channels (Murphy et al., 2011; Jones et al., 2011; Jones et al., 2013a; Jones et al., 2013b). Acidosis caused a rightward shift in steady-state activation, but not in steady-state inactivation in isolated canine ventricular myocytes therefore reducing I_Na,late_ (Murphy et al., 2011).

Many studies have found that hypoxia increases I_Na,late_ (Ju et al., 1996; Carmeliet, 1999; Harnmarstrom and Gage, 2002; Wang et al., 2007; Shimoda and Polak, 2011; Tang et al., 2012). Following a 15 minute hypoxic period, *Wang et al*. reported an increased BM channel activity, a plausible explanation of the increased I_Na,late_.

Intermediary lipid metabolites shown to increase I_Na,late_. Na_v_ channels treated with lysophosphatidylcholine exhibited a sustained BM channel activity (Burnashev et al., 1991; Undrovinas et al., 1992), while palmitoylcarnitine induced a slowly inactivating sodium current (Wu and Corr, 1994). According to more recent data, poly-unsaturated fatty acids (docosahexaenoic acid and eicosapentaenoic acid) reduce both I_Na,early_ and I_Na,late_ (Pignier et al., 2007). According to the authors, the reduction is caused by a decreased overlap between the steady-state activation and inactivation voltage range.

Nitric oxide (NO) has been shown to enhance I_Na,late_ (Ahern et al., 2000). The neural NO synthase (nNOS) belongs to the huge macromolecular complex of Na_v_1.5, with caveolin-3 and α1-syntrophin among some additional proteins (Cheng et al., 2013).

#### Other Mechanisms

##### Transcriptional Regulation

The possible promoter regions and their role in the regulation of human SCN5A gene transcription has already been reported. (Yang et al., 2004; van Stuijvenberg et al., 2010) Recent studies have shown that the zinc-finger transcription factor, GATA4 (Tarradas et al., 2017), and the myocyte enhancing factor-2C (MEF2C) enhances SCN5A transcription (Zhou et al., 2018). However, most likely many other transcription factors are involved in the transcriptional regulation of the SCN5A gene.

##### Glycosylation

Some amino acid motifs found in the Na_v_1.5 protein are subject to N-glycosylation. Carbohydrates account for an about 5 % of the total mass of Na_v_ channels in the rat heart (Cohen and Levitt, 1993). The lack of channel glycosylation caused shifts toward positive voltages in both steady state activation and inactivation curves when naturally sialic-acid deficient channels were used (Zhang et al., 1999), or when these carbohydrate residues were removed by enzymatic treatment (Ufret-Vincenty et al., 2001) Glycosylation also seem to be involved in channel trafficking (Mercier et al., 2015; Cortada and Brugada, 2019)

##### Protein Kinase C

Upon protein kinase C activation, Na^+^ channels are internalized from the plasma membrane (Hallaq et al., 2012). For the process, both channel phosphorylation on S1503 and ROS are required (Liu et al., 2017).

##### Phosphorylation on Tyrosine Residues

The “Fyn” tyrosine kinase phosphorylates Na_v_1.5 channels on the Y1495 Tyr residue, located in the III–IV linker domain. This tyrosine residue helps with anchoring Ca^2+^/CaM to the inactivation gate of the channel (Sarhan and Van Petegem, 2009). When Fyn phosphorylates the channel on Y1495, it increases the window voltage range by shifting the steady-state inactivation toward more positive potentials (Ahern et al., 2005), therefore resulting in an enhanced I_Na,late_.

##### Arginine Methylation

There are three known arginine residues in Na_v_1.5 (R513, R526, and R680), that are subject to methylation (Beltran-Alvarez et al., 2011). These residues are found in the domain I and domain II linker region. There are two known mutations of these arginines (namely R526H and R680H) that cause Brugada (Kapplinger et al., 2010) and LQT3 syndromes (Wang et al., 2007), respectively.

##### Mechanosensitivity

Mechanical stimuli also affect channel gating in Na_v_1.5 channels. *Beyder et al*. investigated this phenomenon both in an expression system (Beyder et al., 2010) and in isolated mouse ventricular cells (Beyder et al., 2012). The pressure ramp applied by the authors caused a 235 % increase in LSM Na_v_1.5 channel openings suggesting that I_Na,late_ is enhanced by mechanical stress. Similar mechanical effects can modify certain signal transduction mechanisms like nNOS and CaMKII (Jian et al., 2014), which can, in turn, increase I_Na,late_.

## The Role of Sodium Homeostasis and Elevated I_Na,late_ in Cardiac Arrhythmias

**Figure 2:**
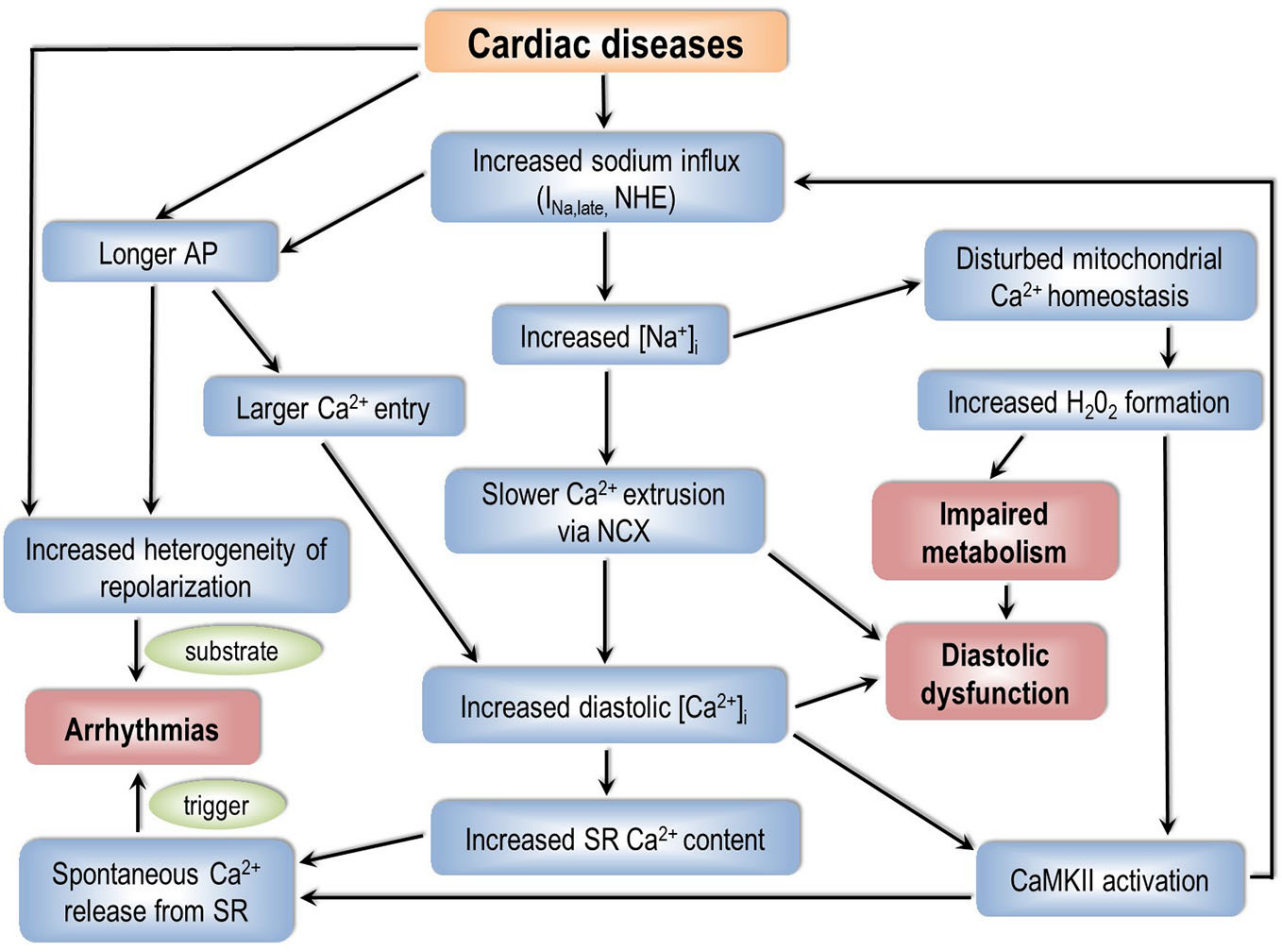
How can an impaired sodium homeostasis of cardiac myocytes lead to arrhythmias? AP, action potential; NHE, Na^+^/H^+^ exchanger; [Na^+^]_i_, intracellular sodium concentration; NCX, Na^+^/Ca^2+^ exchanger; [Ca^2+^]_i_, intracellular calcium concentration; SR, sarcoplasmic reticulum; CaMKII, calcium/calmodulin dependent protein kinase II.

The pathophysiology of cardiac arrhythmias is based on the classical concept of “arrhythmic triad”; combination of a proarrhythmic *substrate*, a *trigger*, and the *modulating effect of the autonomic nervous system* (Merchant and Armoundas, 2012). The exact combination depends on etiology, cardiac-, and extracardiac comorbidities. Abnormal [Na^+^]_i_ homeostasis can play a role in creating an arrhythmia-prone substrate as well as in generating a trigger for the rhythm disorder. The discussed mechanisms are summarized on **Figure 2**.

### [Na^+^]_i_ Increases in Many Cardiac Pathologies

Compared to non-failing myocytes, [Na^+^]_i_ is about 2–6 mM larger in myocytes from failing hearts (Pieske et al., 2002; Despa et al., 2002; Schillinger et al., 2006; Louch et al., 2010). In a pressure- and volume-overload rabbit HF model, Despa et al. have found an increased TTX-sensitive Na^+^ influx (Despa et al., 2002). Interestingly, this larger influx was present not only in electrically stimulated myocytes, but in non-paced cells as well. In paced cells the most plausible candidate of this increased TTX-sensitive Na^+^ influx is I_Na,late_. However, the underlying mechanism of this influx is not yet understood completely in resting myocytes.

### I_Na,late_ Can Contribute to the Elevated [Na^+^]_i_

Many cardiac diseases are associated with an increased I_Na,late_. The list contains cardiac myocytes originating from end-stage HF (Maltsev et al., 1998; Maltsev et al., 2007) and post-myocardial infarction (Huang et al., 2001) preparations as well as animal HF models (Valdivia et al., 2005; Maltsev et al., 2007). The larger I_Na,late_ can be caused by several pathophysiologic factors including oxidative stress (ROS (Song et al., 2006; Sossalla et al., 2008) and NO (Ahern et al., 2000) mainly by S-nitrosylation of the Na_v_1.5 channels (Cheng et al., 2013)), hypoxia (Carmeliet, 1999; Tang et al., 2012), mechanical stress (Beyder et al., 2012), and certain ischemic metabolites, for example oxidized lipids (Burnashev et al., 1991). Looking at gating modes in single Na_v_1.5 channels, enhanced I_Na,late_ is likely underlain by an increased number of BM and LSM openings (Undrovinas et al., 2002; Maltsev, 2006) in HF.

The Ca^2+^—CaM—CaMKII signal transduction pathway is upregulated in HF (Bers, 2010), and this pathway has been shown to increase I_Na,late_ (Tan et al., 2002; Wagner et al., 2006; Ashpole et al., 2012; Ma et al., 2012). Oxidation activates CaMKII (Wagner et al., 2011) and keeps it constitutively active. The enhanced CaMKII-mediated Na_v_1.5 phosphorylation, therefore, certainly takes part in increasing I_Na,late_ under oxidative stress. Recent studies have found that Na_v_1.8 expression is significantly up-regulated, while Na_v_1.5 is reduced in human left ventricular hypertrophy (Ahmad et al., 2019) and HF (Dybkova et al., 2018).

### The Vicious Circle of CaMKII—I_Na,late_—[Na^+^]_i_—[Ca^2+^]_i_—CaMKII

When [Na^+^]_i_ is elevated, it makes the NCX forward mode energetically less favorable, therefore a smaller amount of Ca^2+^ will leave the cell through NCX. This causes an increased [Ca^2+^]_i_ load, and therefore further activates CaMKII, leading to enhanced phosphorylation of CaMKII targets such as Na_v_1.5. This, in turn, increases I_Na,late_, which further elevates [Na^+^]_i_ finally creating an arrhythmogenic vicious circle (Grandi and Herren, 2014). By using genetic (LQT3 mutation) as well as pharmacological (anemone toxin-II, ATX-II) approaches to increase I_Na,late_, and therefore achieve [Na^+^]_i_ loading, Yao et al. described this feedback (Yao et al., 2011). These conditions lead to the vicious circle described above, and as a result, arrhythmias can be generated because of an increase in the CaMKII-dependent phosphorylation of phospholamban and RyRs.

### [Na^+^]_i_—Mitochondrial [Ca^2+^]—Oxidative Stress—CaMKII—I_Na,late_—[Na^+^]_i_ Feedback

The mitochondrial NCX dynamically equilibrate concentrations of Ca^2+^ and Na^+^ of the mitochondrion and the cytosol. Ca^2+^ in the mitochondrion plays a role in determining the production of ATP and ROS by regulating the expression of enzymes involved in oxidative phosphorylation (Yang et al., 2014). If [Na^+^]_i_ is elevated, it will impair Ca^2+^ accumulation in the mitochondrion at high pacing rates, leading to a decrease in NADH/NAD^+^ redox potential. This increases H_2_O_2_ generation in the cells (Liu and O'Rourke, 2008), causing oxidative stress and thereby directly and indirectly (through CaMKII (Erickson et al., 2008)) activating I_Na,late_. Finally, the process leads to a further increase in [Na^+^]_i_ (Wagner et al., 2011). This shows that, similar to an elevated [Na^+^]_i_, CaMKII activation can be caused by and can also lead to an increased ROS production.

### Arrhythmogenic Consequences of an Increased I_Na,late_ and [Na^+^]_i_

Many inherited and acquired diseases can lead to a longer ventricular repolarization, presented as long QT (LQT) syndromes (El-Sherif et al., 2019; Locati et al., 2019). The inherited LQT3 syndrome is caused by an increased I_Na,late_ because of a mutant, much slower inactivating Na_v_1.5 channel. Acquired LQTs include for example heart failure (Maltsev et al., 1998; Maltsev et al., 2007; Coppini et al., 2013), myocardial ischemia and post-infarction state (Huang et al., 2001; Rivera-Fernandez et al., 2016), and type 2 diabetes mellitus (Ninkovic et al., 2016).

Under physiological conditions there is a fine balance between the inward and outward currents during the AP plateau. During the plateau phase the impedance of the membrane is large, therefore even a small change in the delicate balance can lead to a marked change in AP duration. In this setting, the depolarizing drive caused by an increased I_Na,late_ causes a longer AP (Studenik et al., 2001; Horvath et al., 2013), as well as under a longer AP, I_Na,late_ will generate a larger Na^+^ influx. Even in normal hearts, both APD and I_Na,late_ is greater in Purkinje fibers and in “M” cells than in the rest of the myocardium contributing to the physiological heterogeneity of repolarization. LQT syndromes increase both the spatial heterogeneity of repolarization (Maltsev et al., 2007) and the temporal variability of repolarization (El-Sherif et al., 2019) and therefore can present an *arrhythmogenic substrate*. This can be further exaggerated by bradycardia, where the APs are already long, and having larger heterogeneity (Szentandrassy et al., 2015). Cardiac diseases can also provide the *proarrhythmic substrate* in the form of temporal repolarization heterogeneity, “repolarization alternans” (Bonatti et al., 2014; Justo et al., 2016) which phenomenon is more pronounced in tachycardia.

The *trigger* is also highly rate-dependent. At low heart rates, where the cardiac APs are already long even under physiological conditions, an augmented I_Na,late_ can further prolong repolarization therefore increasing the probability of early afterdepolarizations (EADs), and the risk for (fatal) ventricular arrhythmias (Wang et al., 1995; Wang et al., 1996; Makita et al., 2002; Hedley et al., 2009; Cardona et al., 2010; Yamamura et al., 2010; Lowe et al., 2012). Severe bradycardia together with an enhanced I_Na,late_ and a long APD may also promote delayed afterdepolarization (DAD)-mediated triggered activities (Song et al., 2008; Coppini et al., 2013; Horvath et al., 2013). These triggered activities seem to heavily depend on an increased [Ca^2+^]_i_.

As described previously, an increased [Na^+^]_i_ offsets NCX, decreasing Ca^2+^ removal from the cytosol (Bers, 2002; Nagy et al., 2004; Despa and Bers, 2013). This elevates diastolic [Ca^2+^]_i_ and therefore increasing SR Ca^2+^ content; leading to spontaneous Ca^2+^ release events from the Ca^2+^-overloaded SR (Gyorke and Terentyev, 2008). This can generate DADs and therefore possibly triggering arrhythmias. At high heart rates this can further be aggravated by the two feedback loops involving CaMKII, as described in the previous sections, resulting in an enhanced CaMKII mediated phosphorylation of RyR2 therefore increasing the probability of spontaneous SR Ca^2+^ release events. It must be noted again that *in vivo*, there is no high heart rate without β-adrenergic stimulation. Adrenergic stimulation on one hand further activates CaMKII (Hegyi et al., 2018) but on the other hand, it also reduces or even diminishes [Na^+^]_i_ loading of the cells by enhancing NKP activity (Cheung et al., 2010). This makes the role of I_Na,late_ in DAD-mediated arrhythmias occurring at high heart rates questionable.

In the diseased heart, however, rate-dependent properties of I_Na,late_ and [Na+]i are quite poorly investigated. At high pacing rates, I_Na,late_ decreases in LQT3 ΔKPQ mutant cells (Nagatomo et al., 2002) and an increased [Na^+^]_i_ load was reported in hypertrophied feline cells (Mills et al., 2007) as well as in human cardiomyocytes from failing hearts (Pieske et al., 2002).

Pharmacologically enhanced I_Na,late_ increases repolarization heterogeneity in intact, isolated rabbit and guinea pig hearts (Restivo et al., 2004; Milberg et al., 2005), as well as in canine left ventricular wedge preparations giving rise to TdP (Shimizu and Antzelevitch, 1999a; Shimizu and Antzelevitch, 1999b). ATX-II also induces AF in a wide range of experimental conditions (Lu et al., 2012; Liang et al., 2016). Many gain-of-function SCN5A mutations (including LQT3) have been associated with atrial fibrillation (AF) (Benito et al., 2008). Also, in cases of chronic (permanent) AF, larger I_Na,late_ was found (Sossalla et al., 2010; Poulet et al., 2015). These data suggest that enhancement of I_Na,late_ might play a role in generating or maintaining AF most likely because of [Na^+^]_i_ overload dependent Ca^2+^ overload (Nattel and Dobrev, 2012).

## I_Na,late_ As an Antiarrhythmic Therapeutic Target

### Sodium Channel Inhibitors

Natural products of peptide and non-peptide structure can inhibit sodium channels, although these compounds have negligible therapeutical relevance. Clinically relevant small-molecule sodium channel inhibitors include local anesthetics, anticonvulsants, and antiarrhythmic agents such as lidocaine, carbamazepine, phenytoin, lamotrigine, and mexiletine. These small-molecule inhibitors all bind to the so-called “local anesthetic site” of sodium channels where amino acid residues are highly conserved among different Na_v_ subtypes (de Lera Ruiz and Kraus, 2015). Because of this, the “classic” Na_v_ blockers are not subtype specific, they inhibit all subtypes to a certain extent. Also, these compounds somewhat inhibit both I_Na,early_ and I_Na,late_, usually having a higher inhibitory effect on I_Na,late_. Therefore most Na_v_ blockers reduce excitability and impulse propagation (parameters associated with I_Na,early_) together with the plateau sodium current (I_Na,late_).

### Selective I_Na,late_ Inhibitors

A few sodium channel blockers differ from the “classic” inhibitors, because they inhibit I_Na,late_ more potently than I_Na,early_. The molecular mechanism of the preferential I_Na,late_ inhibition is still not completely understood. Even though ranolazine was used for most of the experimental and clinical studies, other selective I_Na,late_ inhibitors also exist such as lidocaine, GS-458967, GS-462808, F15845, and GS-6615 (eleclazine). The half-maximal inhibitory concentration (IC_50_) values of these inhibitors for the late and the early sodium current component are summarized in **Table 1**. For a more thorough data summary on this, see Table 2 in the review of Antzelevitch et al. (2014).

**Table 1:** IC_50_ values of selective late sodium current inhibitors for the late and the early sodium current component.

Compound	IC_50_ for		Reference
	I_Na,late_	I_Na,early_
Lidocaine	29 µM	367 µM	Antzelevitch et al., 2014
Ranolazine	17 µM	1329 µM	Belardinelli et al., 2013
	6 µM	294 µM	Undrovinas et al., 2006
GS-458967	333 nM	<15 % block at 333 nM	Koltun et al., 2016a
	130 nM	7.5 % reduction at 10 µM	Belardinelli et al., 2013
GS-462808	1.9 µM	10 % reduction at 10 µM	Koltun et al., 2016b
GS-6615	0.62 µM	51 µM	Zablocki et al., 2016
F15845	3.25 µM	23 % reduction at 10 µM	Vacher et al., 2009

Where the IC_50_ value is missing, inhibition percentage at a given concentration was used instead.

For lidocaine, IC_50_ values of around 25 and 300 µM were determined for I_Na,late_ and I_Na,early_, respectively (Antzelevitch et al., 2014).

In case of ranolazine, the IC_50_ values are 17 µM for I_Na,late_ and 1,329 µM for I_Na,early_ in rabbit (Belardinelli et al., 2013), whereas 6 µM for I_Na,late_ (Antzelevitch et al., 2004; Undrovinas et al., 2006) and 294 µM for I_Na,early_ (Undrovinas et al., 2006) in canine ventricular myocytes.

GS-458967 was found to have an IC_50_ of 333 nM for I_Na,late_ inhibition while exhibiting smaller than 15% block of I_Na,early_ at the same concentration at 1 and 3 Hz pacing frequencies (Koltun et al., 2016a) measured on Na_v_1.5 channels expressed in HEK-293 cells with automated patch-clamp. In rabbit ventricular cardiomyocytes, the IC_50_ was 130 nM for I_Na,late_, and at 10 µM, GS-458967 caused an approximately 7.5 % reduction in I_Na,early_. (Belardinelli et al., 2013). Unfortunately for the developer, GS-458967 had a high brain penetration and a profound use dependent block on all the various sodium channel isoforms, making the compound prone for possible central nervous system side effects (Koltun et al., 2016a).

GS-462808 has an IC_50_ of 1.9 µM for I_Na,late_ inhibition while blocking 10 % of I_Na,early_ at 10 µM and it is also more cardiac isoform selective than GS-458967 blocking only 8 % of the Na_v_1.1 peak current. The problem with GS-462808 is that it caused liver lesions during the acute animal toxicity tests (Koltun et al., 2016b).

For GS-6615 the IC_50_ values of 0.62 and 51 µM were reported for I_Na,late_ and I_Na,early_ blockade, respectively, in manual patch-clamp experiments, with practically no effect on Na_v_1.1 channels (Zablocki et al., 2016).

F15845 has an IC_50_ of 3.25 µM for the inhibition of veratridine-induced I_Na,late_ while blocking 23 % of I_Na,early_ at 10 µM (Vacher et al., 2009). Last experimental data about F15845 were published in 2010, where it prevented ischemia-induced arrhythmias in rats (Pignier et al., 2010). Since then no new results came out regarding this agent.

Selectivity of these specific I_Na,late_ inhibitors is usually voltage-dependent, these blockers have very little effect on I_Na,early_ at more negative (quite unphysiological, for example −120 mV) holding potentials. As the holding potential gets closer to physiological resting membrane potentials, the selectivity of these compounds decrease, they start to inhibit I_Na,early_ more. Also, most inhibitors block the sodium channels in a rate-dependent (“use-dependent”) fashion; the blockers are more effective at rapid than at slow heart rates. This is because most inhibitors preferentially bind to the open and/or inactivated channels rather than the closed channel. This effect is especially strong in sodium channel blockers having fast association and dissociation kinetics (Pless et al., 2011) (Vaughan-Williams class Ib agents).

In case of 1 µM GS-458967 for example, I_Na,early_ did not change in rabbit ventricular myocytes held at −120 mV at pacing rates of 0.1, 1, or 3 Hz. When the holding potential was −80 mV, however, 1 µM GS-458967 reduced I_Na,early_ by 48 ± 7%, 50 ± 7%, and 56 ± 8% at rates of 0.1, 1, and 3 Hz, respectively (Belardinelli et al., 2013).

Ranolazine also inhibits sodium channels in a voltage-, and use-dependent fashion, moreover this blockade is also significantly larger in atria compared to ventricles (Zygmunt et al., 2011). With 50 ms long depolarizing pulses and 250 ms diastolic intervals (at 3.33 Hz), the use-dependent block by ranolazine at −120 mV was 21 % in ventricular, versus 32 % in atrial cells, whereas at −100 mV the block was 47 % versus 56 %, respectively. This data suggest that the rate dependency (use-dependency) is very pronounced in case of I_Na,early_ inhibition, but much smaller with I_Na,late_. Therefore, based on the rate-dependent physiological (see *Heart Rate and AP Duration Influences I_Na,late_*) and pharmacological characteristics of I_Na,_ _early_ and I_Na,late_, a quite selective inhibition of I_Na,late_ might be achieved at slow heart rates and with long APs, but at fast rates, with short AP duration, sodium channel blockers similarly inhibit both I_Na,_ _early_ and I_Na,late_.

At therapeutical plasma concentrations, ranolazine inhibits other ionic currents besides I_Na,late_. This includes I_Kr_ (approximately 40 % inhibition at 6 µM), and I_Ca,L_ (around 25 % inhibition at 6 µM) (Antzelevitch et al., 2004). Consequently, inhibiting I_Na,late_ and applying ranolazine are very far from being identical concepts. When ranolazine is used to inhibit I_Na,late_, effects on other channels must not be forgotten. Besides the previous features, ranolazine is also a weak β-adrenergic antagonist (Letienne et al., 2001) and an inhibitor of fatty acid oxidation (Chaitman, 2006), even though this latter effect only becomes prominent at supratherapeutical plasma concentrations.

### Non-Cardiac Sodium Channel Inhibitors as Potential Antiarrhythmic Agents

Riluzole blocks TTX-sensitive sodium channels preferentially, which are associated with damaged neurons (Song et al., 1997). Riluzole also directly inhibits the kainate and NMDA receptors (Debono et al., 1993) as well as potentiates GABA_A_ receptors (He et al., 2002). In anaesthetized pigs, myocardial damage and arrhythmias induced by coronary occlusion has been reduced by riluzole (Weiss et al., 2013). Riluzole has also been found to be anti-ischemic and antiarrhythmic in a pig model of acute myocardial infarction. (Weiss and Saint, 2010).

Targeting Na_v_1.8 with specific inhibitors might provide a potential novel approach in the future in antiarrhythmic drug therapy, because recent studies have found that Na_v_1.8 expression is significantly up-regulated in human left ventricular hypertrophy (Ahmad et al., 2019) and HF (Dybkova et al., 2018). By using Na_v_1.8-specific blockers [either A-803467 (30 nM) or PF-01247324 (1 μM)] the authors managed to reduce I_Na,late_ and APD in these experiments. Other Na_v_1.8 specific inhibitors include funapide and VX-150, however these compounds have not been tested in relation to cardiac pathophysiology so far.

### Experimental Pathophysiology Studies

Because of the pronounced use-dependent effect of specific I_Na,late_ inhibitors, interpretation of experimental studies conducted on rats and mice (having resting heart rates around 400 bpm) are very difficult. Therefore this review will focus on experimental data originating from larger mammalian species.

#### Late Sodium Current Inhibition and Ventricular Arrhythmias

As it was demonstrated in the previous sections, I_Na,late_ has quite different characteristics under different heart rates. Therefore it is worthwhile to split the ventricular arrhythmia topic into two subtopics accordingly.

##### Bradycardia and Long APs

Many *in vitro* experimental studies have shown that at low pacing rates with prolonged APs and increased repolarization heterogeneity (LQT3 syndrome, heart failure, hypertrophic cardiomyopathy), inhibition of I_Na,late_ effectively reduces the burden of arrhythmic episodes [EADs, DADs, triggered APs, Torsade de Pointes (TdP) (Shimizu and Antzelevitch, 1997; Song et al., 2004; Coppini et al., 2013; Belardinelli et al., 2013; Rajamani et al., 2016)].

Ranolazine and GS-458967 has been shown to suppress dofetilide-induced TdP in a canine in vivo model (Antoons et al., 2010; Bossu et al., 2018).

There was one experimental study about the potential antiarrhythmic role of F15845, where it prevented ischemia-induced arrhythmias in rats (Pignier et al., 2010). However the use of a rat model makes it hard to extrapolate this study to humans.

Under the pathological conditions listed above, the fine balance between the inward and outward currents during the AP plateau is shifted toward the depolarizing inward currents, resulting in a longer AP. Therefore, theoretically, any intervention that reduces the depolarizing currents (e.g.: L-type calcium current, NCX current, I_Na,late_) could be effective in bringing the repolarization closer to normal. In this setting, therefore, inhibiting I_Na,late_ will reduce the depolarization drive resulting in a significantly shorter APD and the suppression of arrhythmogenic events such as EADs, even if the magnitude of I_Na,late_ is not increased. Under similar conditions, other interventions such as L-type calcium channel blockade (Abrahamsson et al., 1996) or potassium channel activation (Carlsson et al., 1992) can also shorten APD, reduce repolarization heterogeneity, and suppress the occurrence of arrhythmogenic events even if I_Na,late_ is upregulated. In LQT syndromes I_Na,late_-mediated increase in Ca^2+^_i_ is just a fraction of the total Ca^2+^_i_, and even total Ca^2+^_i_ just contributes to rather than determines the arrhythmogenic events (Carlsson et al., 1996).

##### Tachycardia-Induced Tachyarrhythmias (VT, VF)

I_Na,late_ blockers seem to effectively prevent or terminate tachycardia-induced ventricular tachycardia, and ventricular fibrillation in healthy animal models in the presence of a β-adrenergic agonist (Alves Bento et al., 2015; Carneiro et al., 2015; Bacic et al., 2017).

However, inhibition of I_Na,late_ does not likely play a crucial role here, based on the following theoretical considerations. To start with, in healthy ventricular tissue at high heart rates I_Na,late_ is quite small, as it was discussed in *Heart Rate and AP Duration Influences I_Na,late_*. Furthermore, at rapid heart rates with β-adrenergic stimulation the major arrhythmogenic mechanism is likely to be the increased L-type calcium current, the increased SR Ca^2+^ content, and the leaky RyR together (Merchant and Armoundas, 2012). The third but similarly important factor is that these VT/VF episodes are likely underlain by a reentry mechanism, therefore heavily depending on the fast conduction provided by I_Na,early_. At high pacing rates the “specific” I_Na,late_ inhibitors will also block a considerable amount of I_Na,early_ as well (see *Selective I_Na,late_ Inhibitors* for details), and this might just be enough to break the reentry circuit (Burashnikov and Antzelevitch, 2017).

Based on the experimental data, I_Na,late_ inhibition seems to be a valid therapeutic approach to tackle ventricular arrhythmias especially at low heart rates. These experimental studies also suggest that I_Na,late_ inhibition mainly affects the arrhythmogenic substrate by making the repolarization less heterogenous (Carneiro et al., 2015), with only low impact on suppressing the triggers (Bossu et al., 2018).

#### Late Sodium Current Inhibition in AF

GS-458967 was shown to suppress isoproterenol-, and high Ca^2+^-induced DADs in healthy canine pulmonary-, and superior vena cava preparations (Sicouri et al., 2013). GS-458967 also suppressed autonomically triggered AF in an intact porcine model (Carneiro et al., 2015). In other experimental studies, “classic” sodium channel inhibitors (eg, lidocaine, flecainide) also prevented and terminated AF (Wang et al., 1992; Comtois et al., 2008). However, these agents were used at concentrations causing a suppression of I_Na,early_. Experimental data about ranolazine shows an effective reduction of AF burden (AFB) only at concentrations that potently inhibit both I_Na,early_ (Burashnikov et al., 2007; Kumar et al., 2009; Burashnikov et al., 2014) and I_Kr_ (Burashnikov et al., 2007) Suppressing I_Kr_ reduces the diastolic interval between APs therefore promoting rate-dependent I_Na,early_ inhibition.

Based on these data, *specific* I_Na,late_ blockade alone is not a clear and straightforward approach in AF, except for cases when a longer atrial AP is the pathogenetic factor in the initiation of AF.

### Clinical Studies

#### Ranolazine

So far, ranolazine has been used in the vast majority of clinical studies involving I_Na,late_ blockers. When interpreting these trials, it has to be considered that ranolazine has other effects besides inhibiting I_Na,late_. With the use of ranolazine, the first favorable results from phase 2 clinical trials were published in the 1990s (Cocco et al., 1992; Thadani et al., 1994). In 2006, following the outcome of the MARISA (Chaitman et al., 2004a), CARISA (Chaitman et al., 2004b), and ERICA (Stone et al., 2006) trials, the Food and Drug Administration approved ranolazine as an anti-anginal agent.

The effect of clinical outcome and safety of ranolazine therapy was investigated in more than 6,500 patients with non-ST-elevation acute coronary syndrome in the MERLIN TIMI-36 trial (Morrow et al., 2007). Although cardiovascular death or myocardial infarction has not been significantly reduced by ranolazine when compared to standard therapy; but recurrent ischemia (Morrow et al., 2007) and the incidence of arrhythmias (Scirica et al., 2007) were significantly lower with ranolazine. Treatment with ranolazine, compared to placebo, resulted in significantly lower incidences of arrhythmias. Fewer patients had episodes of ventricular tachycardia lasting more than eight beats [166 (5.3%) versus 265 (8.3%); p<0.001], supraventricular tachycardia [1413 (44.7%) versus 1752 (55.0%); p<0.001], or new-onset AF [55 (1.7%) versus 75 (2.4%); p=0.08]. Moreover, longer than 3 s pauses were less frequent with ranolazine [97 (3.1%) versus 136 (4.3%); p=0.01].

In the double-blind HARMONY (ClinicalTrials.gov ID: NCT01522651) phase 2 trial (Reiffel et al., 2015), patients with paroxysmal AF and implanted pacemakers were enrolled, so that AFB could continuously be monitored over the 12 weeks of treatment period. Patients were randomized to placebo, ranolazine alone (750 mg twice a day—BID), dronedarone alone (225 mg BID), or ranolazine (750 mg BID) combined with dronedarone (either 150 mg BID or 225 mg BID). The idea behind the combination was to reduce the dose of dronedarone, and therefore the negative inotropic effect associated with dronedarone. Placebo or the drugs alone did not significantly reduce AFB. In the combination therapies, however, ranolazine with dronedarone 225 mg BID reduced AFB by 59% vs placebo (p=0.008), and ranolazine with dronedarone 150 mg BID reduced AFB by 43% (p=0.072). Also, patients tolerated both combinations well.

Into the RAFFAELLO phase 2 trial (De Ferrari et al., 2015) patients with persistent AF (7 days to 6 months) were enrolled. Two hours after successful electrical cardioversion participants were randomized to either placebo, or ranolazine 375 mg, 500 mg, or 750 mg BID. Patients were monitored daily by transtelephonic ECG. The primary end-point was the time to first AF recurrence. No dose of the ranolazine prolonged time to AF recurrence significantly compared to placebo. Of the 238 patients who took at least one dose of the study drug, AF recurred in 56.4%, 56.9%, 41.7%, and 39.7% of patients in the placebo and ranolazine 375 mg/500 mg/750 mg groups, respectively. The reduction in overall AF recurrence in the combined 500-mg and 750-mg groups was of borderline significance compared to the placebo group (p=0.053) and significant compared to 375-mg group (p=0.035).

The RAID clinical trial (NCT01534962) (Zareba et al., 2018) investigated high-risk cardiomyopathy patients who received an implantable cardioverter-defibrillator (ICD). The subjects received either ranolazine (1,000 mg BID) or placebo. The primary endpoints were VT or VF requiring ICD shock or death. Among 1,012 ICD patients the ranolazine versus placebo hazard ratio was 0.84 (95% confidence interval: 0.67 to 1.05; p=0.117) for the primary endpoint. In the ranolazine group the risk of ICD therapies for recurrent VT or VF were significantly lower (hazard ratio: 0.70; 95% confidence interval: 0.51 to 0.96; p=0.028). Other effects of ranolazine treatment however has not been significant. These included individual components of the primary endpoint, quality of life, cardiac hospitalizations, and inappropriate ICD shocks as well.

In a smaller group of participants of the RAND-CFR trial (NCT01754259) (Evaristo et al., 2018) where symptomatic diabetic patients participated with non-flow-limiting coronary artery stenosis with diffuse atherosclerosis and/or microvascular dysfunction, effect of ranolazine on T-wave heterogeneity was evaluated. At physical rest, in the ranolazine group T-wave heterogeneity was 28 % smaller (placebo: 47±6 μV, ranolazine: 39±5 μV, p=0.002), however ranolazine did not differ from the placebo group during exercise. The trial also suggested that reduction in repolarization abnormalities seemed to be independent of alterations in myocardial blood flow.

In a meta-analysis of eight randomized clinical trials (Gong et al., 2017) *Gong et al*. found that ranolazine significantly reduced AF incidence in different clinical settings, such as in acute coronary syndromes, after cardiac surgery and after electrical cardioversion of AF (relative risk=0.67, 95% confidence interval: 0.52–0.87, p=0.002). Moreover, the combined use of ranolazine and amiodarone compared to amiodarone alone showed a 1.23-times higher conversion rate of AF (95% confidence interval: 1.08–1.40), together with a significantly, about 10 h shorter conversion time.

Based on the evidence above, ranolazine may have therapeutic role in the treatment of cardiac rhythm disorders, of both atrial and ventricular origin. For stronger evidence, more phase 3 clinical investigations are necessary.

#### Eleclazine (GS-6615)

Besides ranolazine, until now eleclazine was the only other selective I_Na,late_ inhibitor drug candidate that made it to phase 3 clinical trials. In the first trial (NCT02300558) eleclazine was tested for safety, tolerability, and its effect on shortening of the QT interval in LQT3 patients. The primary outcome of the study showed that after 24 weeks the mean daytime corrected QT interval was significantly, 8.5 ms shorter than at baseline, and there was only one patient with a serious adverse event (nephrolithiasis). The other trial (LIBERTY-HCM; NCT02291237) targeted HCM patients for the effect of eleclazine on exercise capacity. In this trial, eleclazine has not been proven to be superior to placebo.

The last moment in the development of eleclazine came after results of the phase 2 TEMPO study (NCT02104583) were analyzed. In the trial, subjects with ventricular tachycardia/ventricular fibrillation and ICD participated. Results of the study have shown that the rate of ICD shocks was higher in subjects who received eleclazine compared to the placebo arm. Therefore in late 2016, the further development of eleclazine was terminated for all indications.

## Conclusions

An increased I_Na,late_ is present in many heart diseases. The upregulated I_Na,late_ lengthens the cardiac AP, increases [Na^+^]_i_, and causes Ca^2+^ overload of cardiomyocytes by offsetting the forward mode NCX. The elevated Ca^2+^, in turn, mainly through CaMKII, can further increase I_Na,late_ in a vicious circle. These pathophysiological mechanisms together may result in impaired cardiac energetics and contractile dysfunction of the heart as well as cardiac arrhythmias. The prolonged AP can serve as a substrate that is prone to rhythm disorders, whereas Ca^2+^ overload can be the trigger. I_Na,late_ seems to possess a pathogenetic role especially in AF and in ventricular arrhythmias occurring under bradycardic conditions.

Multitude of pathophysiology studies have drawn the consequence that selective I_Na,late_ inhibition is a favorable antiarrhythmic tool in many experimental settings. Despite all these studies, the one and only drug on the market that selectively inhibits I_Na,late_ is ranolazine, although it significantly affects other ionic currents as well. Ranolazine has been a safe and effective antianginal medication since 2006 based on large randomized studies. Some recent clinical evidence also proves that ranolazine shows favorable effects in AF and in ventricular arrhythmias. For stronger evidence, more phase 3 clinical investigations are necessary. Targeting Na_v_1.8 with specific inhibitors is also an interesting novel approach in the future of antiarrhythmic drug therapy.

## Author Contributions

BH: conception, design and drafting the manuscript TH, DK, KK: writing sections of the manuscript JM, PN, TB: conception and final review of the manuscript. All authors agreed on publishing the manuscript in the current form.

## Funding

Funding was obtained from the National Research, Development and Innovation Fund for research projects FK-128116 and PD-120794, and the Thematic Excellence Programme ED_18-1-2019-0028. Further funding was obtained from the GINOP-2.3.2-15-2016-00040 and EFOP-3.6.2-16-2017-00006 projects, which are co-financed by the European Union and the European Regional Development Fund. Research of BH and DK was supported by the Ministry of Human Capacities (ÚNKP-19-4-DE-284 to BH, ÚNKP-19-3-II-DE-288 to DK). The work was also supported by the Hungarian Academy of Sciences (János Bolyai Research Scholarship to BH).

## Conflict of Interest

The authors declare that the research was conducted in the absence of any commercial or financial relationships that could be construed as a potential conflict of interest.
